# Prevalence of Anti-Synthetase Syndrome in Patients of Interstitial Lung Disease with Connective Tissue Diseases and Autoimmune Features: A Cross-Sectional Study

**DOI:** 10.31138/mjr.180324.dtc

**Published:** 2025-03-31

**Authors:** Indu MB, Desh Deepak, Ajay Bhatta, Gunjan Lalwani, Brijesh Sharma, Vardhini Somayya, Mala Chhabra, Nandini Duggal

**Affiliations:** 1 Department of General Medicine, Atal Bihari Vajpayee Institute of Medical Sciences & Dr Ram Manohar Lohia Hospital, New Delhi, India;; 2 Department of Pulmonary Medicine, Atal Bihari Vajpayee Institute of Medical Sciences & Dr Ram Manohar Lohia, New Delhi, India;; 3 Department of Microbiology, Atal Bihari Vajpayee Institute of Medical Sciences & Dr Ram Manohar Lohia, New Delhi, India

**Keywords:** anti-synthetase syndrome, interstitial lung disease, connective tissue diseases, interstitial pneumonia with autoimmune features, anti-aminoacyl tRNA synthetase antibodies

## Abstract

**Objective:**

Anti-synthetase syndrome (ASS) is a rare autoimmune disease with heterogenous manifestations. Interstitial lung disease (ILD) is one among its common manifestations. The aim of this study was to evaluate the prevalence of ASS in cases of ILD associated with autoimmune features and describe the clinical, serological, and radiological profile in them.

**Methods:**

This cross-sectional study included a total of 100 patients: 50 cases each of connective tissue disease-related ILD (CTD-ILD) and interstitial pneumonia with autoimmune features (IPAF).

**Results:**

Four cases of CTD-ILD and 7 cases of IPAF had anti-ARS auto-antibodies. All eleven of them fulfilled Connor’s criteria for ASS. The classic triad of arthritis, myositis, and ILD was present only in two cases. Anti-nuclear antibody (ANA) was positive in 63.6%. Anti-Jo1 (54.56%), Anti-PL12(27.3%), anti-PL7(18.2%), and anti-EJ(18.2%) were the anti ARS autoantibodies. Though generally considered to be mutually exclusive, anti-PL12 and anti-EJ antibodies were found together in two cases. Myalgia was associated with all four ARS antibodies. Anti-Jo1 antibody was associated with Raynaud’s phenomenon, polyarthralgia, polyarthritis, and myopathy. Anti-PL7 antibody was associated with myopathy and mechanic’s hands. Anti-PL12 and anti-EJ antibodies were associated with inflammatory poly-arthritis, polyarthralgia, and unexplained fever. Non-specific interstitial pneumonia (NSIP) was the most common radiologic pattern of ILD (81.8%). The remaining two had Usual interstitial pneumonia (UIP) pattern and were positive for anti-Jo1 antibody.

**Conclusion:**

ASS can present in many ways, often incomplete at the onset without the classic clinical triad. Anti-ARS autoantibodies can be found in established CTDs. Anti-cytoplasmic antibodies (not ANA) must be used to screen for ASS in suspected cases.

## INTRODUCTION

Anti-synthetase syndrome (ASS) is a rare autoimmune systemic disorder with heterogeneous clinical manifestations. Anti-aminoacyl tRNA synthetase (ARS) autoantibodies are the hallmark of this entity. Interstitial lung disease (ILD) occurs in 71–100% cases of ASS and it may be the sole clinical manifestation at the disease onset in 15% cases of ASS.^1–3^ It is the major determinant of morbidity and mortality in ASS cases.^4^ Moreover, 68% of ASS cases with positive myositis-specific antibodies develop ILD in the course of the disease.^5^ In this study, we sought anti-ARS autoantibodies in cases of interstitial lung diseases associated with autoimmune features.

## MATERIALS AND METHODS

This was a 5-year (2018 to 2023) single-centre cross-sectional observational study conducted in the Department of Medicine at a tertiary care centre in New Delhi. The study protocol was approved by the institutional ethics committee (TP(MD/MS)(127/2018)/IEC/PGIMER/RMLH; 24-10-2018) and written informed consent was obtained from all the participants. The study was conducted in accordance with the principles of Declaration of Helsinki. The study followed the Strengthening the Reporting of The Observational Studies in Epidemiology (STROBE) reporting guidelines. We screened consecutive cases of High resolution computed tomography(HRCT) proven ILD above the age of 18 years who were referred to our clinic. A sample size of 96 would be sufficient to observe 7% of anti synthetase syndrome in interstitial lung disease with 20% relative precision and 95% confidence interval.^6,7^ 50 cases that fulfilled criteria for a connective tissue disorder(American College of Rheumatology/European League Against Rheumatism) and 50 cases of interstitial pneumonia with autoimmune features (IPAF) (as per European Respiratory society and American Thoracic Society criteria) were included in this study. Their previous medical reports were analysed. Demographics, relevant clinical data, blood and serological investigations, and radiological patterns of ILD were studied. Anti-ARS antibodies were sought for in these cases.

ANA test was done by enzyme-linked immunosorbent assay (ELISA) and extractable nuclear antigen and myositis profile by immunoblot assay. The myositis panel in our study centre included ASS specific antibodies like anti-Jo1, anti-PL7, anti-PL12, and anti-EJ; myositis-specific ASS unrelated autoantibodies like anti-SRP, anti-MDA5, anti-Mi2, anti-TIFI γ, and myositis-associated antibodies like anti-Ro52, anti-Ku, anti-PmScl, and anti-U1RNP. The autoantibody testing for other ARS antibodies anti-OJ, anti-KS, anti-Zo, and anti-Ha were not available at our study centre.

Connor’s criteria were used to diagnose ASS in this study.

Connor et al. proposed that a diagnosis of ASS can be made in the presence of anti ARS autoantibody with one or more of the following clinical features: a) mechanic’s hands; b) Raynaud’s phenomenon; c) myositis; d) ILD; e) arthritis; and f) unexplained fever.^8^

## RESULTS

The study included 100 subjects: 50 cases of connective tissue disease-related ILD (CTD-ILD) and 50 cases of IPAF. The mean age of the study population was 46.12 years(range 21 to 75 years). The female-to-male ratio in this study was 3.5:1.

The study included cases of ILD associated with systemic sclerosis, rheumatoid arthritis, Sjögren’s syndrome, mixed connective tissue disorder, inflammatory myopathies like dermatomyositis and polymyositis, psoriasis, systemic lupus erythematosus, overlap syndrome, and IPAF (**Table 1).**

**Table 1.** Frequency distribution of rheumatological diseases in this study and distribution of anti ARS autoantibodies.

**Sl. No**	**Rheumatological Diagnosis**	**Frequency (n = 100)**		**Frequency of anti ARS antibodies (n = 11)**	
		**%**	**N**	**%**	**N**
**A**	**Rheumatologic Diseases**	**50%**	**50**	**36.36%**	**4**
1	Systemic sclerosis	24	24	18.18%	2
2	Rheumatoid arthritis	7	7	-	-
3	Sjögren's syndrome	6	6	-	-
4	Systemic lupus erythematosus	2	2	9.1%	1
	Idiopathic Inflammatory myopathy	3	3	9.1%	1
5	Dermatomyositis		2		
	Polymyositis		1		
6	Mixed connective tissue disorders	2	2	-	-
7	Overlap syndrome	5	5	-	-
8	Psoriasis	1	1	-	-
**B**	**IPAF**	**50**	**50**	**63.64%**	**7**

Anti-ARS antibodies were present in 11 cases in this study. All eleven cases with anti-ARS autoantibodies satisfied the ASS criteria by Connor’s et al.^8^ Thus, the prevalence of ASS in this study was 11%. The mean age of the ASS group was 47.09 years. The female-to-male ratio was 7:4.

Anti-nuclear autoantibody was positive only in seven (63.6%) of them. ACA reports were available only in one patient. Anti-Jo1 auto-antibody (54.5%) was the most common anti-ARS auto-antibody in this study. The other anti-ARS auto-antibodies were anti-P12 (27.3%), anti-PL7 (18.2%), and anti-EJ auto-antibodies (18.9%). Anti-PL12 and anti-EJ autoantibodies were found together in two cases in this study.

The ASS-unrelated myositis-specific autoantibodies were anti-Mi 2 (6.6%), anti-MDA-5 (3.3%), and anti-SRP (1.1%) antibodies. Anti-Mi 2 and anti-MDA5 antibodies were seen in the non-ASS group, and anti-SRP antibody was seen in the ASS group. The myositis-associated autoantibodies (MAA) were anti-Ro-52, anti-Ku, anti-U1RNP, and anti-Pm-Scl. These occurred with varying frequencies in both ASS and non-ASS groups. The frequency distribution of the myositis-related autoanti-bodies in this study is given in **Table 2**.

**Table 2.** Myositis related autoantibodies in this study (n =100).

**Auto antibody**	**ASS group**		**Non-ASS group**	
	**Antibody**	**n(%) n=11**	**Antibody**	**n(%) n=89**
**ASS related Myositis specific autoantibodies**	Anti Jo 1	6(6.6)%	None	
	Anti PL 7	2(2.2)%		
	Anti PL 12	3(3.3)%		
	Anti E	2(2.2)%		
**ASS unrelated Myositis specific auto antibodies**	Anti SRP	1(1.1)%	Anti Mi 2	6(6.7)%
			Anti MDA 5	3(3.4)%
**Myositis associated autoantibodies**	Anti Ro 52	5(5.5)%	Anti Ro 52	30(34)%
	Anti Ku	2(2.2)%	Anti Ku	8(9)%
	Anti U1RNP	3(3.3)%	Anti U1RNP	12(12.4)%
	Anti Pm Scl	1(1.1)%	Anti Pm Scl	9(9)%

Two cases of localised scleroderma and a case each of polymyositis and systemic lupus erythematosus with secondary Sjögren’s syndrome had anti ARS auto-antibodies. Respiratory symptoms developed in them during the course of the disease. Hence, a revised diagnosis of ASS-overlap syndrome was made in all four of them. Seven (63.64%) cases of IPAF tested positive for anti-ARS autoantibodies and a revised diagnosis of ASS was considered in them.

The main clinical features in the ASS group are given in **Table 3.** Breathlessness during exertion was the presenting manifestation in all of them. The most common extrapulmonary manifestations in this study were myalgia(27.3%), proximal muscle weakness (18.2%), arthralgia (18.2%), inflammatory polyarthritis(18.2%),

**Table 3.** Demographic, serological, clinical, and radiological profile in the ASS group.

**Case**	**Age/Sex**	**Initial diagnosis**	**Muscle enzyme**	**ANA**	**ENA**	**Anti ARS antibody against**	**Other myositis specific antibody against**	**Other myositis related antibody against**	**Clinical features (Extra pulmonary)**	**HRCT pattern**
1	59/M	LSSc	Not raised	+	U1RNP Ku	Jo 1	-	Ku U1RNP	Raynaud's phenomenon Skin thickening	UIP
2	25/F	LSSc	Not raised	+	Anti Scl 70 Anti centromere	Jo 1	-	-	Raynaud's phenomenon Skin thickening	NSIP
3	50/M	IPAF	Not raised	-	Ro 52 U1RNP	PL 12 EJ	SRP	Ro 52 U1RNP	Unexplained fever Myalgia Polyarthralgia	NSIP
4	37/M	Pm	Raised	+	-	PL 7	-	-	Polyarthralgia Proximal muscle weakness^*^	NSIP
5	46/M	IPAF	Raised	+	Ro 52 U1RNP	Jo 1	-	Ro 52 U1RNP	Polyarthralgia Proximal muscle weakness^*^	NSIP
6	47/F	IPAF	Raised	+	Ro 52	Jo 1	-	Ro 52	Myalgia Inflammatory Polyarthritis	NSIP
7	25/F	IPAF	Raised	+	Ku Ro 60	PL EJ PL 12	-	Ku	Myalgia Inflammatory polyarthritis	NSIP
8	50/F	with 2° SLE SjS	Not raised	+	Ro 52 ds DNA Histone	Jo 1	-	Ro 52	Dryness of eyes, mouth	UIP
9	60/M	IPAF	Not raised	-	-	Jo 1	-	-	None	NSIP
10	59/F	IPAF	Raised	-	Ro 52 Pm Scl	PL 7	-	Ro 52 Pm Scl	Mechanic's hands	NSIP
11	60/F	IPAF	Not raised	-	Ku	PL 12	-	Ku	Unexplained fever	NSIP

* Both cases had biopsy proven myositis; Polymyositis in Case 4 and inflammatory myositis in Case 5.

**Figure 1.**
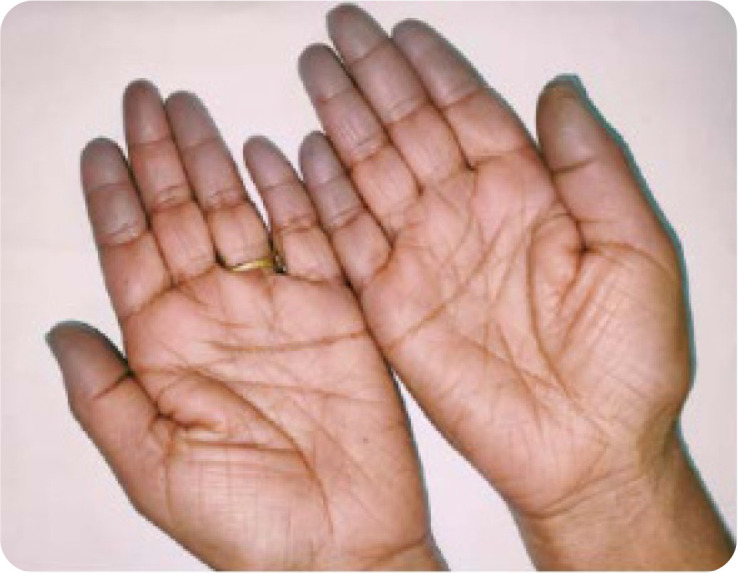
Raynaud’s phenomenon showing bluish discolouration of fingertips on exposure to cold (demarcation of colour difference shown).

**Figure 2.**
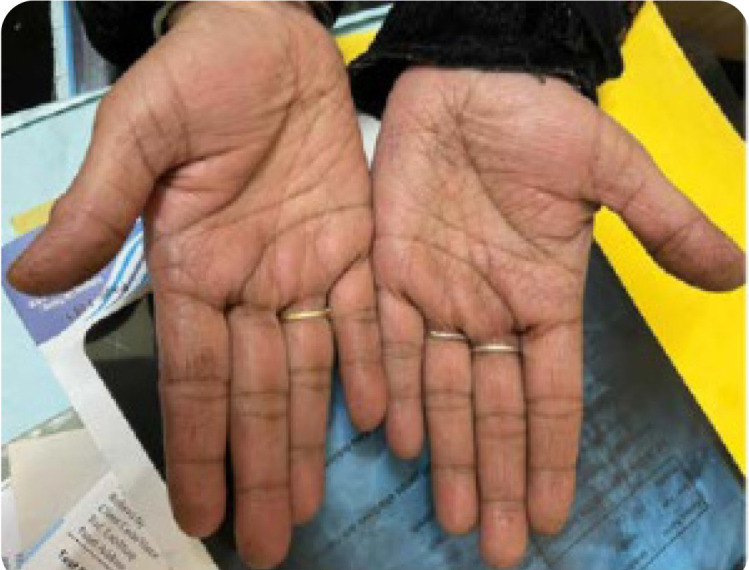
Mechanic’s hands showing fissuring and cracking of hands mostly in the lateral aspect.

Raynaud’s phenomenon (18.2%) (**Figure 1**), unexplained fever(18.2%), and mechanic’s hands (9.1%) (**Figure 2**). The two cases with Raynaud’s phenomenon had ASS/Localised scleroderma overlap. One case with anti-Jo1 antibody related ASS had only respiratory symptoms- ILD was the sole manifestation.

All the four anti ARS antibodies were associated with myalgia in this study. Proximal myositis was seen with both anti-Jo1 and anti-PL7 antibodies. However, muscle enzymes (creatine phosphokinase) were elevated in five of the eleven cases. Inflammatory polyarthritis and polyarthralgia was seen with anti-Jo1, anti-PL12, and anti-EJ antibodies. Raynaud’s phenomenon was seen in two cases with anti-Jo1 anti-ARS antibody. Unexplained fever was seen in cases with anti-PL12 and anti-EJ antibodies. The only case with mechanic’s hand was positive for anti-PL7 antibody.

The anti-SRP autoantibody was the only myositis-specific, ASS unrelated autoantibody in the ASS group. The patient with the anti-SRP antibody also had anti-PL12 and anti-EJ antibodies. This case had myalgia, arthralgia, and an unexplained fever with normal muscle enzymes.

The most common myositis-associated autoantibodies in this study were anti-Ro52 (45.6%), anti-U1RNP (27.9%), anti-Ku (18.9%) and anti-PmScL (9.1%). Both anti-Ro52 and anti-U1RNP autoantibodies were most commonly associated with the anti-Jo1 ARS antibody.

**Figure 3.**
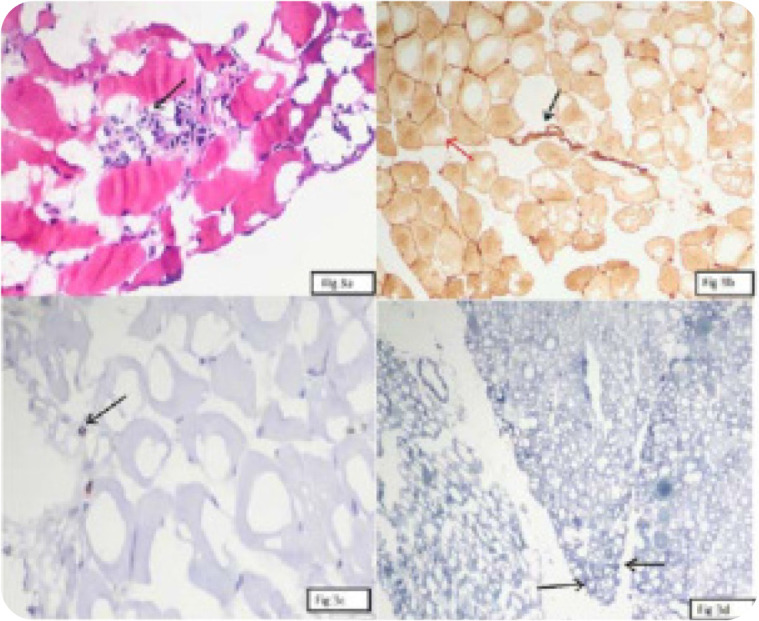
Muscle biopsy in ASS case. **(a)** Myonecrosis with myophagocytosis. **(b)** HLA staining positive in sarcolemma with perifascicular accentuation. **(c)** Immunohistochemistry showing CD3 positive inflammatory cells. **(d)** NADH staining showing perifascicular atrophy.

The classic triad of ILD, myositis, and arthritis was present in only two cases in this study. All three features of the triad were present at the onset of illness in one patient. The other patient presented with polymyositis and developed ILD and arthritis during the course of illness. Both of them had biopsy-proven myositis (**Figure 3A–D**), and both fulfilled Solomon’s and Connor’s criteria for ASS. Only the one with anti-Jo1 antibody associated ASS fulfilled the American College of Rheumatology/European League Against Rheumatism(ACR/EULAR)2017 criteria.

**Figure 4.**
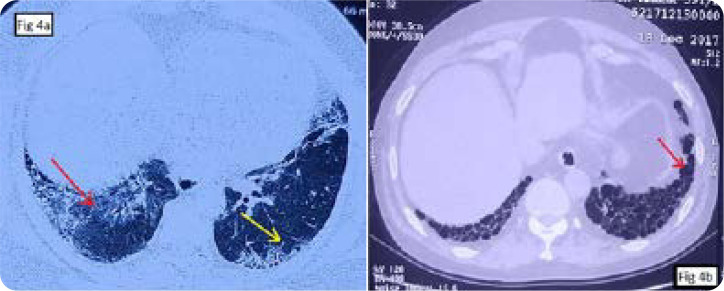
HRCT chest in ASS. **(a)** Ground glassing (red arrow) and interstitial thickening (yellow arrow) in non-specific interstitial pneumonia. **(b)** Honey combing (red arrow) in usual interstitial pneumonia.

The most common HRCT pattern in cases of ASS was non-specific interstitial pneumonia (NSIP) in 9 (81.8%), followed by usual interstitial pneumonia (UIP) in the remaining two (18.2%) (**Figure 4A–B**). Anti-Jo1 antibody was the anti-ARS antibody seen in both cases of UIP pattern.

## DISCUSSION

ASS was first described by Marguerie et al. in 1990 and was described as a triad of polymyositis, diffuse ILD, and serum autoantibodies to aminoacyl tRNA synthetases (ARS).^9^ ASS has a prevalence of 0.6 per 100,000 people.^10^ The mean age at diagnosis is 50 years, and females are twice as likely to be affected as males.^10–12^ The female predisposition and mean age at onset in middle ages were evident in this study as in prior studies.

ARS are cytoplasmic enzymes that initiate protein translation. Anti-ARS autoantibodies are the hallmark of ASS.^13^ The two most commonly used criteria to diagnose ASS include Connor’s criteria and Solomon’s criteria.^8,14^ Anti-ARS autoantibodies are the cornerstone for the diagnosis of ASS in both these criteria. The heterogeneity in the clinical spectrum of ASS is attributed to them. Eight anti-ARS autoantibodies have been identified to date, namely anti-Jo1, anti-PL7, anti-PL12, anti-EJ, anti-OJ, anti-KS, anti-Zo, and anti-Ha. ILD is one of the clinical features in both these criteria. However, a third criterion the EULAR/ACR diagnostic criteria consider only one of the anti-ARS antibodies i.e. anti-Jo1 autoantibody^15^ (**Table 4**).

**Table 4.** Diagnostic criteria for ASS.

	**Connors et al.**	**Solomon et al.**	**ACR/EULAR 2017**
Anti ARS antibody (must)	Any	Any	Anti Jo 1 only
Clinical criteria	Any one of: ILD Myositis (Peter Bohan criteria) Unexplained fever Raynaud's phenomenon Mechanic's hands Arthritis	Major criteria 1.ILD 2. Polymyositis/Dermatomyositis by Peter and Bohan criteria Minor criteria Raynaud's phenomenon Mechanic's hands Arthritis	Muscle weakness Skin lesions Age of onset Elevated muscle enzymes Muscle biopsy
Diagnosis of ASS made if	Anti ARS plus any one of the six clinical criteria	Anti ARS antibody plus either Two major criteria or One major plus two minor	Anti Jo 1 antibody plus score of 7.5 without muscle biopsy (8.7 with muscle biopsy)
Number of cases in this study satisfying ASS criteria	11 (100%)	2 (18.2%)	1(9.1%)

In our study all the 11 cases with anti-ARS antibodies met the Connor’s et al. criteria for ASS, as all of them had ILD. There were two cases with proximal myopathy (both biopsy proven)- one with anti-Jo1 antibody and the other with anti-PL7 antibody. Both of them also met the Solomon et al. criteria for ASS.

We had chosen Connor’s criteria for the diagnosis of ASS in this study as it had higher sensitivity and comparable specificity, compared to Solomon criteria.^16^ ACR/EULAR 2017 criteria was not chosen as it take into consideration only Anti-Jo1 antibody and it does not give importance to other Anti-ARS antibodies. Also the latter criteria is more applicable for patients with IIMs.

Autoantibodies in ASS can be anti-nuclear or anti-cytoplasmic. Anti-ARS autoantibodies are directed against cytoplasmic targets. Anti-cytoplasmic antibodies (ACA) were significantly higher in ASS patients than ANA. ACA showed high sensitivity, specificity, negative predictive value and accuracy for anti-ARS antibody positivity than ANA.^17^ Thus, a negative ANA does not rule out ASS because the antigenic targets are cytoplasmic.

The most common among these are anti-Jo-1, anti-EJ, anti-PL7, and anti-PL12 antibodies, with 68–87% of them being anti-Jo1 antibodies.^18,19^ The common anti-ARS antibodies in this study also had the same trend. Anti-ARS autoantibodies are generally considered to be mutually exclusive, yet co-occurrences have been described.^20,21^ Both anti-PL12 and anti-EJ autoantibodies were found together in two cases in this study.

The most common extrapulmonary manifestations in this study were myalgia (27.3%), proximal muscle weakness (18.2%), arthralgia (18.2%), inflammatory polyarthritis (18.2%), Raynaud’s phenomenon (18.2%), unexplained fever (18.2%), and mechanic’s hands (9.1%).

The myriad clinical presentations in ASS are attributed to the anti-ARS antibody. The classical triad of ILD, myositis, and arthritis is found in only 19.5% of cases at disease onset.^5^ The classic triad was present only in two cases (19.1%) in this study. Of the two cases with the classic triad, one had all the three features at the onset and the other developed them during the disease course. The frequency of classic complete forms of ASS increases from 19% at the disease onset to 50% with the disease progression.^5^ The classical forms of ASS are usually seen with anti-Jo1, anti-OJ, anti-EJ, anti-Zo, and anti-Ha ARS antibodies.^18^ The anti-ARS autoantibodies associated with the classic forms of ASS in this study were anti-Jo1 and anti-PL7 antibodies.

Patients with anti-Jo1 antibodies can develop ILD in up to 90% of cases. In a cohort study by American and European network of ASS, among anti Jo 1 positive patients, 50% had ILD at the disease onset and 84% developed the ILD during follow up.^22^ Among the six patients with anti Jo 1 antibodies in this study, 3 had ILD at the disease diagnosis and the rest 3 developed ILD during the disease course. ILD associated with anti-Jo1 antibodies is less symptomatic and causes less fibrosis.^23,24^ However, both the cases of UIP pattern of ILD in this study was associated with anti Jo 1 antibody.

ILD is the most common isolated manifestation of ASS in patients with anti-PL7, anti-PL12, and anti-EJ antibodies.^18,24^ ILD in anti-PL7 antibody related ASS is milder than that in anti-PL12 antibody related ASS. All the three patients with anti-PL12 antibody (of which two had anti-EJ along with anti-PL12) had ILD at the disease onset. In the two cases with anti-PL7 antibody; one had ILD as the initial manifestation and the other developed ILD during the disease course. Acute onset of ILD (like organising diffuse alveolar damage) was observed in 74.1% of patients with anti-EJ antibodies.^12^ Both cases of anti-EJ antibody related ASS in this study had ILD at diagnosis but neither of them had an acute presentation.

The HRCT pattern in ASS-related ILD cases was NSIP in 39–72.5%, OP in 14.6–43%, and UIP in 9.76% of cases.^12^ The NSIP pattern was the most common HRCT pattern of ILD in this study (82%) and was seen with all four anti-ARS antibodies. However, both the cases of UIP(18%) pattern on HRCT was associated with anti-Jo1 antibody.

The spectrum of muscular involvement in ASS ranges from isolated elevation of muscle enzymes to marked muscle weakness and immobility. Myalgia without muscle weakness is also observed.^25^ Five patients had muscular symptoms in this study, two had muscle weakness and the other three had myalgia. Muscle enzymes – creatine phosphokinase was elevated only in five of the 11 cases (45.5%). Muscle symptoms are less in patients with anti- PL7 or PL12 antibodies as compared with anti-Jo1 antibody.^23^ Myalgia was associated with anti-Jo1, anti-PL12, and anti-EJ antibodies in this study. Proximal muscle weakness was seen in patients with anti-Jo1 and anti-PL7 antibodies. Proximal muscle weakness with myositis preceded ILD in the anti-PL7-related ASS in this study.

Arthritis and arthralgia are common signs in ASS, with a prevalence ranging from 20–88%.^26^ Symmetrical polyarthritis involving small joints of the hands is more common in ASS.^9^ Arthritis occurs more frequently in the anti-Jo1 antibody related ASS as compared to the other ASS subtypes. Isolated arthritis can be the initial symptom of the disease in 24% of patients with anti-Jo-1 antibodies.^10^ Polyarthralgia was associated with all four anti-ARS antibodies in this study. Inflammatory polyarthritis was one of the initial manifestations in two cases- one with anti-Jo1 related ASS (as a part of classic triad) and the one with co-existence of anti-PL12 and anti-EJ antibodies.

The less specific clinical signs in this study were Raynaud’s phenomenon and unexplained fever. The prevalence of Raynaud’s phenomenon in ASS is highly inconsistent, varying from 8.7 to 65%. In a study by Canagava et al., Raynaud’s phenomenon was the initial symptom in 15% of anti-Jo-1-related ASS.^5^ Raynaud’s phenomenon is reported to be more common in patients with joint symptoms than those with myositis or ILD.^27^ Both cases of Raynaud’s phenomenon in this study were ASS/localised Systemic sclerosis overlap and were positive for anti-Jo-1 antibodies. Certain studies describe scleroderma resembling the pattern of nail fold capillaroscopy in long-standing ASS cases or those with anti-Jo-1 antibodies, but a correlation of this with Raynaud’s phenomenon is yet to be proven. The mechanic’s hand describes hyperkeratosis over the palms, predominantly in the distal and lateral aspects of the phalanges. The roughening and cracking of the skin results in irregular, dirty-appearing lines that resemble mechanic’s hands.^28^ The prevalence of this key sign in ASS cases ranges from 36.6% to 38 %.^12^ These are common in patients with anti-ARS autoantibodies- especially anti-Jo-1 antibodies than others. The single case of mechanic’s hands in this study was associated with anti-PL7 antibody.

The prevalence of unexplained fever ranges from 25.5 to 60.9% in ASS [13]. This was present in two cases in this study. In a study by Canagawa et al., this symptom was commonly associated with anti-PL12 antibody.^24^ Both ASS patients with unexplained fever in this study were also positive for anti-PL12 antibodies, of which one had co-occurrence of anti-EJ antibody.^23^

Anti-signal recognition particle (SRP) antibody was the only myositis-specific ASS-unrelated autoantibody in this study. It is highly specific for immune-mediated necrotising myopathy.^29^ The case with anti-SRP positivity in this study had no features of myopathy and had normal muscle enzymes.

There is an important association between anti-Ro52 and anti-Jo1 antibodies. Anti-Ro52 are considered the most common type of MAA, occurring in 30-65% of cases of ASS.^27^ Anti-Ro52 was the most common MAA in this study and was commonly seen in association with anti-Jo1 antibodies. There is an important association between anti-Ro52 and anti-Jo1, which may be initially confused with Sjögren’s disease, especially when associated with sicca symptoms. Anti-Ro52 has been independently associated with ASS and when present can cause more severe and fibrotic ILD.^30^ Hence, a minor salivary gland biopsy is indicated to differentiate between the two. A case of anti-Jo1 antibody associated with anti-Ro 52 antibody in this study had sicca symptoms and was SLE with secondary Sjögren’s syndrome/ASS overlap.

Anti-PmScl is commonly associated with scleromyositis or sclerodermatomyositis.^12^ This antibody was associated with mechanic’s hands in this study. Patient did not have any muscular symptoms but had raised muscle enzymes.

## CONCLUSION

ASS is a rare disease with little literature available on it. The clinical spectrum of ASS is heterogenous. The uniqueness of the clinical spectrum is attributed to the type of anti-ARS autoantibodies. This study, though limited because of availability of only 4 out of 8 anti-ARS antibodies showed varying permutations and combinations of these antibodies that reflected in the clinical profile.

Incomplete manifestations of ASS may develop other manifestations over a variable period of time. Symptoms such as myalgias and polyarthralgias in idiopathic ILDs should be monitored for progression at regular intervals with muscle enzymes and clinical examination. Many cases of ASS may have muscular symptoms without an elevation of muscle enzymes. Thus, patients of ASS are frequently diagnosed as having undifferentiated connective tissue diseases, interstitial pneumonia with autoimmune features, idiopathic ILD, or other CTD-ILD if not tested for these antibodies. Anti-cytoplasmic autoantibodies and not ANA must be used to screen for ASS in suspected cases. Anti-ARS antibodies should be considered in all cases of ILD with positive ACA, autoimmune features, refractory cases of arthritis and myositis, and in ILD cases with mechanic’s hands.

## FUNDING

No funds or grants were obtained for this study.

## CONFLICTS OF INTEREST

The authors declare no conflicts of interest.
